# Co-expression of anti-miR319g and miRStv_11 lead to enhanced steviol glycosides content in *Stevia rebaudiana*

**DOI:** 10.1186/s12870-019-1871-2

**Published:** 2019-06-24

**Authors:** Monica Saifi, Sneha Yogindran, Nazima Nasrullah, Umara Nissar, Irum Gul, M. Z. Abdin

**Affiliations:** 1 Centre for Transgenic Plant Development, Department of Biotechnology, School of Chemical and Life Sciences, JamiaHamdard, Hamdard Nagar, New Delhi, 110062 India; 2 School of Life Sciences, Jawaharlal Nehru University, New Delhi, 110067 India

**Keywords:** *Stevia rebaudiana*, Steviol glycosides, miRNA, Anti-miRNA, miRNA interference, Secondary plant metabolites

## Abstract

**Background:**

miRNAs are major regulators of gene expression and have proven their role in understanding the genetic regulation of biosynthetic pathways. Stevioside and rebaudioside-A, the two most abundant and sweetest compounds found in leaf extract of *Stevia rebaudiana*, have been used for many years in treatment of diabetes. It has been found that the crude extract is more potent than the purified extract. Stevioside, being accumulated in higher concentration, imparts licorice like aftertaste. Thus, in order to make the sweetener more potent and palatable, there is a need to increase the intrinsic concentration of steviol glycosides and to alter the ratio of rebaudioside-A to stevioside. Doing so would significantly increase the quality of the sweeteners, and the potential to be used on a wider scale. To do so, in previous report, miRNAs associated with genes of steviol glycosides biosynthetic pathway were identified in *S. rebaudiana.* In continuation to that in this study, the two miRNAs (miR319g and miRStv_11) targeting key genes of steviol glycosides biosynthetic pathway were modulated and their impact was evaluated on steviol glycosides contents.

**Results:**

The over-expression results showed that miRStv_11 induced, while miR319g had repressive action on its target genes. The knock-down constructs for miR319g and miRStv_11 were then prepared and it was demonstrated that the expression of anti-miR319g produced inhibitory effect on its target miRNA, resulting in enhanced expression of its target genes. On the other hand, anti-miRStv_11 resulted in down-regulation of miRStv_11 and its target gene. Further miRStv_11 and anti-miR319gwere co-expressed which resulted in significant increase in stevioside (24.5%) and rebaudioside-A (51%) contents.

**Conclusion:**

In conclusion, the role of miR319g and miRStv_11 was successfully validated in steviol gycosides biosynthetic pathway gene regulation and their effect on steviol gycosides contents. In this study, we found the positively correlated miRNA-mRNA interaction network in plants, where miRStv_11 enhanced the expression of *KAH* gene. miRNAs knock-down was also successfully achieved using antisense precursors. Overall, this study thus reveals more complex nature and fundamental importance of miRNAs in biosynthetic pathway related gene networks and hence, these miRNAs can be successfully employed to enhance the ratio of rebaudioside-A to stevioside, thus enhancing the sweetening indices of this plant and making it more palatable.

**Electronic supplementary material:**

The online version of this article (10.1186/s12870-019-1871-2) contains supplementary material, which is available to authorized users.

## Background

Understanding of molecular mechanisms of gene regulation has been revolutionarily advanced by the discovery of micro RNA (miRNA) [20]. miRNAs are non-coding regulatory RNAs of ~ 18-24 nt in length that regulate the expression of numerous eukaryotic genes at the post-transcriptional level. These molecules function by preferentially binding to the 3′-untranslated region (3′-UTR) of their target mRNAs. For the functional consequences, 7–8 nt of 5′-end of the miRNA must have exact complementarity to the target mRNA, the so called ‘seed’ region, and partial complementarity with rest of the sequence. Modern genomic approach has documented the importance of miRNAs in regulating biosynthetic pathways related gene networks [6, 29]. miRNAs play an important role in understanding the genetic regulation of biosynthetic pathways, and thus help in manipulation and selection to get better plant genotypes with improved secondary metabolites and increased biomass.

*Stevia rebaudiana,* an important perennial herb, accumulates high concentrations of steviol glycosides [35, 36]. Stevioside and rebaudioside-A, the two most abundant compounds found in leaf extract of this plant, have been used for many years in traditional treatment of diabetes by stimulating insulin secretion. Thus, these compounds seem to be promising natural sweetener with antidiabetic activity [37]. There are several products commercially available that utilize stevia extract or steviol gycosides as a sugar substitute. For example, Coca Cola life is one of the 45 products produced by Coca Cola that use stevia as a sweetener [19]; Pepsi True is PepsiCo’s stevia based soft drink etc. Furthermore, various studies have been conducted that indicate steviol gycosides show no toxicity in mammals [1, 33]. There are more than 30 glycosides present in *S. rebaudiana* such as dulcosideA, rebaudioside A-E, steviolbioside and stevioside etc. The sweetest among these is rebaudioside-A, while stevioside is less sweet than reb-A. These compounds are reported to be around 500 and 200–300 times sweeter than common sucrose, respectively [9, 10, 37]. Stevioside is however, responsible for a bitter aftertaste, sometimes referred to as a liquorice aftertaste. In wild type varieties of stevia, it is found in a higher concentration than rebaudioside-A. Hence, in order to make the sweetener more potent and palatable, there is a need to increase the intrinsic concentration of rebaudioside-A, and reduce the concentration of stevioside. Doing so would significantly increase the quality of the stevia sweetener, not to mention the potential to be used on a wider scale. In our previous study [28], we for the first time identified miRNAs associated with genes of steviol gycosides biosynthetic pathway in *S. rebaudiana.* Among all the miRNAsanalysed for their expression levels, we found the two miRNAs (miR319g and miRStv_11) which showed the expression patterns similar to their target mRNAs. On the other hand, the other miRNAs were found to have expression pattern opposite to their target mRNAs [28]. Thus, it became quite interesting to understand how these two miRNAs (miR319g and miRStv_11) are involved in regulation of steviol glycosides biosynthetic pathway genes. Therefore, in the present study, for functional characterization of these miRNAs, we over-expressed them in leaves of *S. rebaudiana* using *Agrobacterium* mediated transient transformation method. Here, we uncovered the striking positive correlation patterns between up-regulated expression of miRStv_11 and its target gene, *KAH*. On the other hand, miR319g showed the repressive action on its target genes (*KO, KS* and *UGT85C2)*. Further, in order to re-validate the function of miR319g and miRStv_11, we prepared the knock-down constructs for both using an antisense precursor of their target miRNAs. The expression of these antisense miRNAs viz. anti-miR319g and anti-miRStv_11 resulted in suppression of their target miRNAs, miR319g and miRStv_11, which further resulted in de-repression and suppression of their target genes *KO, KS, UGT85C2* and *KAH,* respectively. The results obtained by knocking down miR319g and miRStv_11 thus supports our data of over-expression study. We then co-expressed miRStv_11 and anti-miR319g in leaves of *S. rebaudiana* in order to enhance steviol glycosides contents and found significant increase in stevioside (24.5%) and rebaudioside-A (51%) contents. In this study, we revealed the more complex nature and fundamental importance of miRNAs in biosynthetic pathway related gene networks and hence, these miRNAs can be successfully employed to enhance the ratio of rebaudioside-A to stevioside, so enhancing the sweetening indices of this plant and making it more palatable.

## Results

### miR319g and miRStv_11 mediated negatively and positively correlated miRNA-mRNA regulatory networks

**Fig. 1:**
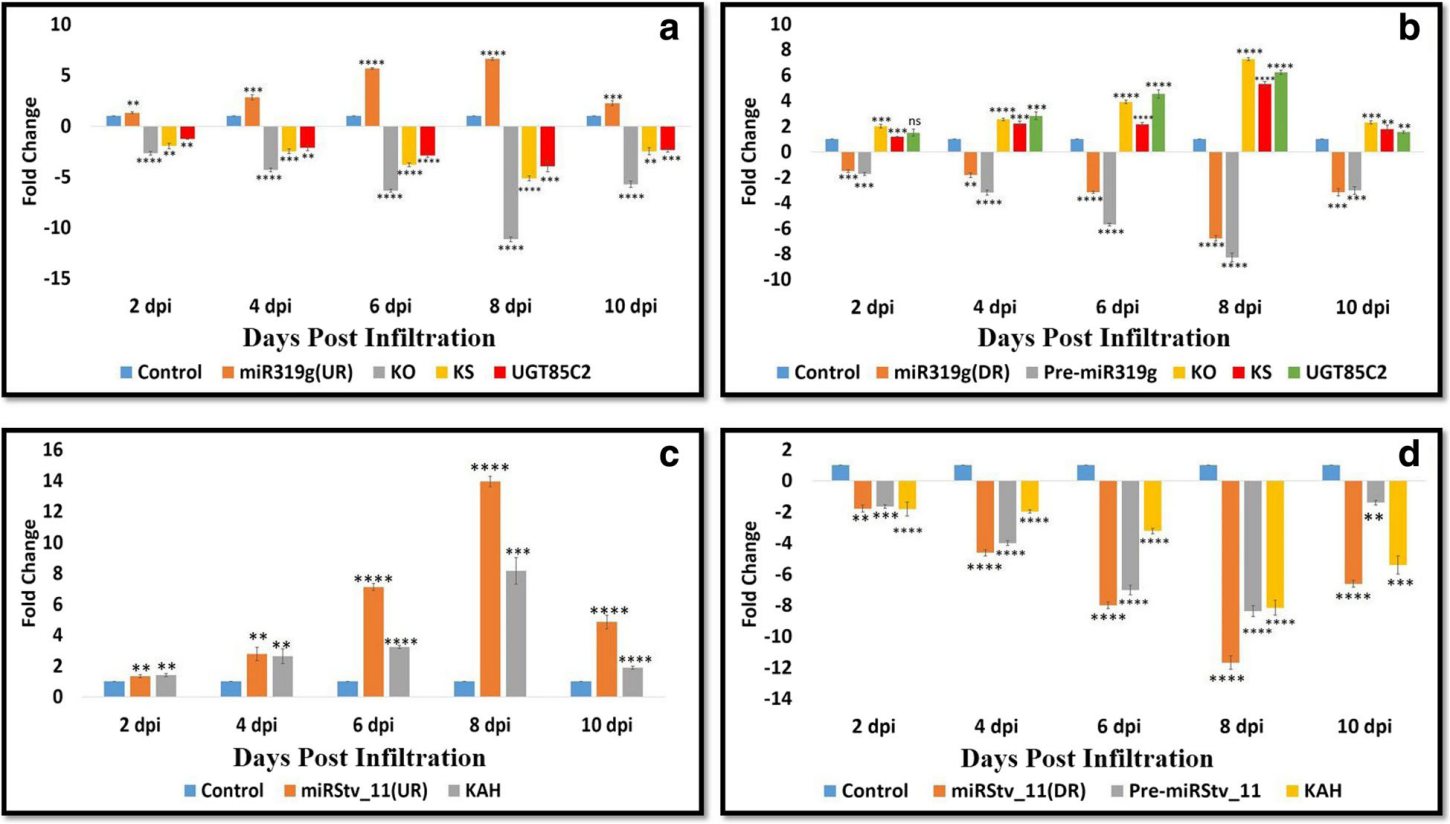
Real-Time PCR analysis of **(a)** miR319g and its target genes when leaves were transformed with miR319g over-expression construct; **b**miR319g and its target genes when leaves were transformed with miR319g down-regulation construct; **c** miRStv_11 and its target genes when leaves were transformed with miRStv_11 over-expression construct; **d** miRStv_11 and its target genes when leaves were transformed with miRStv_11 down-regulation constructs. Expression was normalized to that of actin. miRNAs and their target gene levels from the wild type were set as the control. Error bars indicate ±SE (*n* = 3), (*) indicates significance of variance (1*-0.05; 2*-0.01; 3*-0.001; 4*-0.0001). *UR: Up-regulation. *DR: Down-regulation

For functional characterization of miR319g and miRStv_11 their over-expression constructs were prepared. For which, the precursor sequences of 196 and 320 bp, were amplified, cloned and sequenced for miR319g and miRStv_11, respectively. The sequence of miR319g obtained was found to be 79% identical to the sequence of miR319g from *Populus trichocarpa*, while the miRstv_11 sequence was 99% identical to the EST sequence of *S. rebaudiana* already submitted in NCBI database (Additional file 5). The structures were confirmed by mfold. The precursor sequences of miR319g and miRStv_11 from *S. rebaudiana* generated fold back structures exhibiting branched loop structures. Mature sequences of miR319g and miRStv_11 were located at 3′ arm. The minimum free energy (Kcal/mol) associated with the stem loop structure of miR319g and miRStv_11 were − 66.40 Kcal/mol and − 71.60 Kcal/mol, respectively (Additional files 1 and 2). qPCR analysis was conducted to check the effectiveness of overexpression. The leaves transformed with overexpression construct of miR319g was found to increase mature miR319g expression level up to 6.5 fold than the control resulting in decreased level of its target mRNAs, viz.*KO* (*Kaurene oxidase), KS (Kaurene synthase)* and *UGT85C2 (UDP-glycosyltransferase 85C2)* up to 11, 5 and 3 fold, respectively in due course of agroinfiltration from 2 to 10 days post infiltration (dpi) (Fig. 1a). On the other hand, the construct corresponding to miRStv_11 showed up to 13 fold increase in mature miRStv_11 expression resulting in increased level of its target mRNA i.e. *KAH (Kaurenoic acid-13-hydroxylase)* up to 8 fold in due time period of agroinfiltration (Fig. 1c). Here, we uncovered the striking positive correlation pattern between up-regulated expression of miRStv_11 and its target gene *KAH.*

### miRStv_11 showed high affinity for promoter region of *Kaurenoic acid hydroxylase* gene

**Fig. 2:**
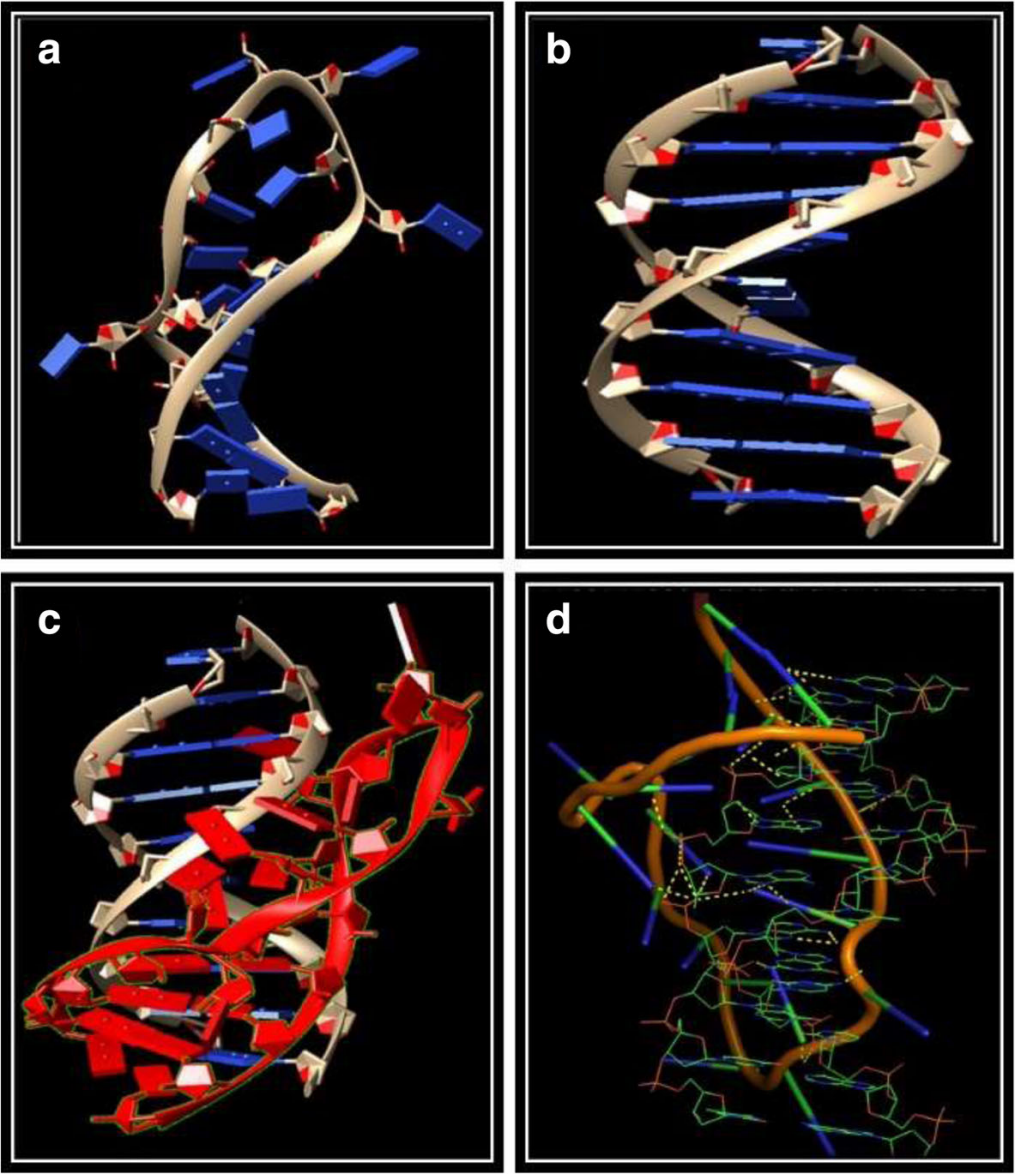
Modeled three dimensional structure of (**a**) miRStv_11; **b** miRStv_11 target site B-DNA structure (*KAH* gene promoter region); **c** Docked complex of miRNA (shown in red color) and miRNA binding sites (shown in yellow color); **d** Atomic level interactions of miRNA and miRNA binding sites

For docking of miRStv_11 with promoter region of *Kaurenoic acid hydroxylase (KAH)* gene, the three dimensional structures of miRStv_11 and *KAH* gene promoter site were modeled (Fig. 2 a and b) and saved into the PDB format. Further, the PDB files of the two modeled biomolecules and their interaction sites were uploaded into PatchDock sever (miRNA Interacting nucleotide atoms: 1, 2, 3, 5B, 13, 14, 16, 17; *KAH* gene promoter interacting nucleotide atoms: 2, 3, 5, 6, 8, 9, 10). The miRNA was observed to bind in the major groove of promoter region of *KAH* gene (Fig. 2c). As miRNA is a heavy bio-molecule, therefore, it must bind the major groove of the receptor DNA. It was interesting to note that the desolvation energy (ACE) for the given receptor binding site had a high negative value (− 937.59). This reveals the high feasibility of this reaction and strong binding with the binding site. A total of 13 hydrogen bonds were found to be involved in the miRNA-DNA interaction, which involves 10 miRNA bases and 9 DNA bases (Additional file 4: Figure S2d).

### Silencing of miR319g and miRStv_11 by their antisense precursors

To re-validate the function of miR319g and miRStv_11, their silencing was done by expressing respective antisense precursors in leaves of *S. rebaudiana*. The leaves of *S. rebaudiana* transformed with knock-down construct corresponding to anti-miR319g was found to reduce mature miR319g expression level up to 6.7 fold resulting in increased level of its target mRNAs, *KO* (*Kaurene oxidase), KS (Kaurene synthase)* and *UGT85C2 (UDP-glycosyltransferase 85C2)* up to 7, 5 and 6 fold, respectively in due course of agroinfiltration from 2 to 10 days post infiltration (dpi) (Fig. 1b). On the other hand, the construct corresponding to anti-miRStv_11 showed up to 11.6 fold decrease in the level of mature miRStv_11 expression resulting in decreased expression level of its target gene, *KAH (Kaurenoic acid-13-hydroxylase)* i.e. up to 8.1 fold in due time period of agroinfiltration (Fig. 1d). To check whether antisense precursors of miR319g and miRStv_11 also interfere at the precursor level, the expression analysis of the precursors of miR319g and miRStv_11 was also done and it was found to be down-regulated up to 8.2 and 8.3 fold, respectively in due time period of agroinfiltration (Fig. 1). This data suggest that the expression of antisense precursors of miR319g and miRStv_11 could down-regulate their target miRNAs and thus affecting the expression of their respective target genes, implicating miRNA interference.

### Specific thermodynamic profiles of miRNA correlates well with its strand selection

**Fig. 3:**
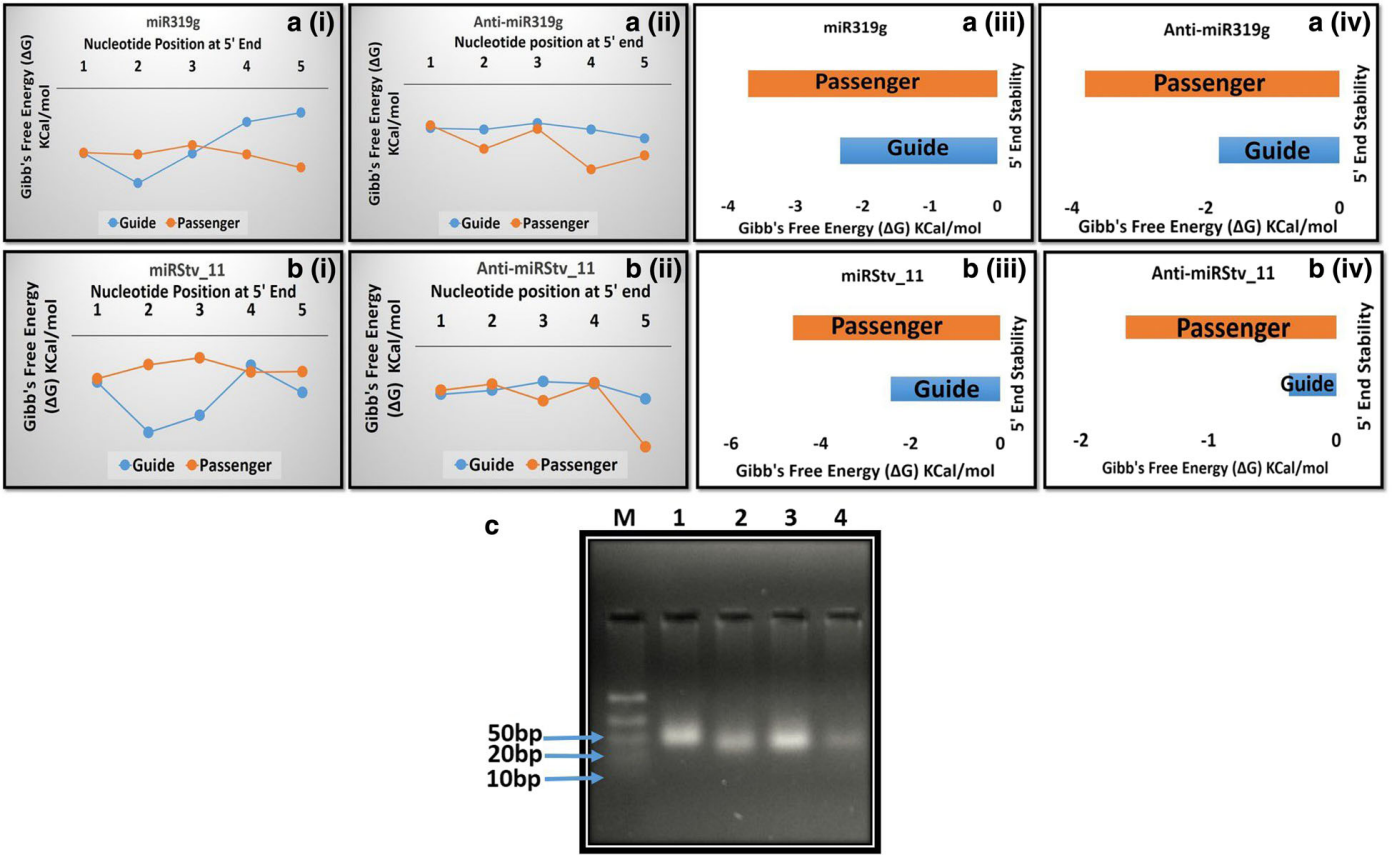
Calculated average internal stability profiles of 5′ end of guide and passenger strands of miRNAs **(a)** at different nucleotide positions for (i) miR319g (ii) anti-miR319g (iii) miRStv_11 (iv) anti-miRStv_11; **b** of hexameric sequences for (i) miR319g (ii) anti-miR319g (iii) miRStv_11 (iv) anti-miRStv_11; **c** confirmation of selection of miR319g (Lane 1), miRStv_11 (Lane 2), anti-miR319g (Lane 3) and anti-miRStv_11 (Lane 4) strands in leaves of *S. rebaudiana,* transformed with knockdown constructs by the presence of ~ 50 bp bands by stem-loop RT-PCR; Lane M 10 bp ladder

To support our experimental data of miRNA interference, it is important to check that which of the strands of miRNA-miRNA* duplex was being selected by the RISC. It has been reported that miRNA strand, whose 5′ end is more weakly bound to the complementary strand, is more readily incorporated into RISC [30]. Thus, it is tempting to calculate the thermodynamic profiles of sense and anti-sense strands of miRNAs as well as anti-miRNAs to predict the functional strand among two. Internal stability, i.e. 5′ end instability of hexamer sub sequences of sense strands of miR319g and miRStv_11 as well as anti-miR319g and anti-miRStv_11 was found lower (− 2.3, − 2.8 and − 1.3, − 1.9 Kcal/mol) than the anti-sense strands (− 3.7, − 4.8 and − 3.6, − 3.2 Kcal/mol), thus making them functional or guide strands (Fig. 3). Selection of miR319g, miRStv_11, anti-miR319g and anti-miRStv_11 strands in leaves of *S. rebaudiana,* transformed with knockdown constructs were confirmed by the presence of ~ 50 bp bands by stem-loop RT-PCR (Fig. 3c).

### Effect of co-expression of anti-miR319g and miRStv_11 on their target genes

**Fig. 4:**
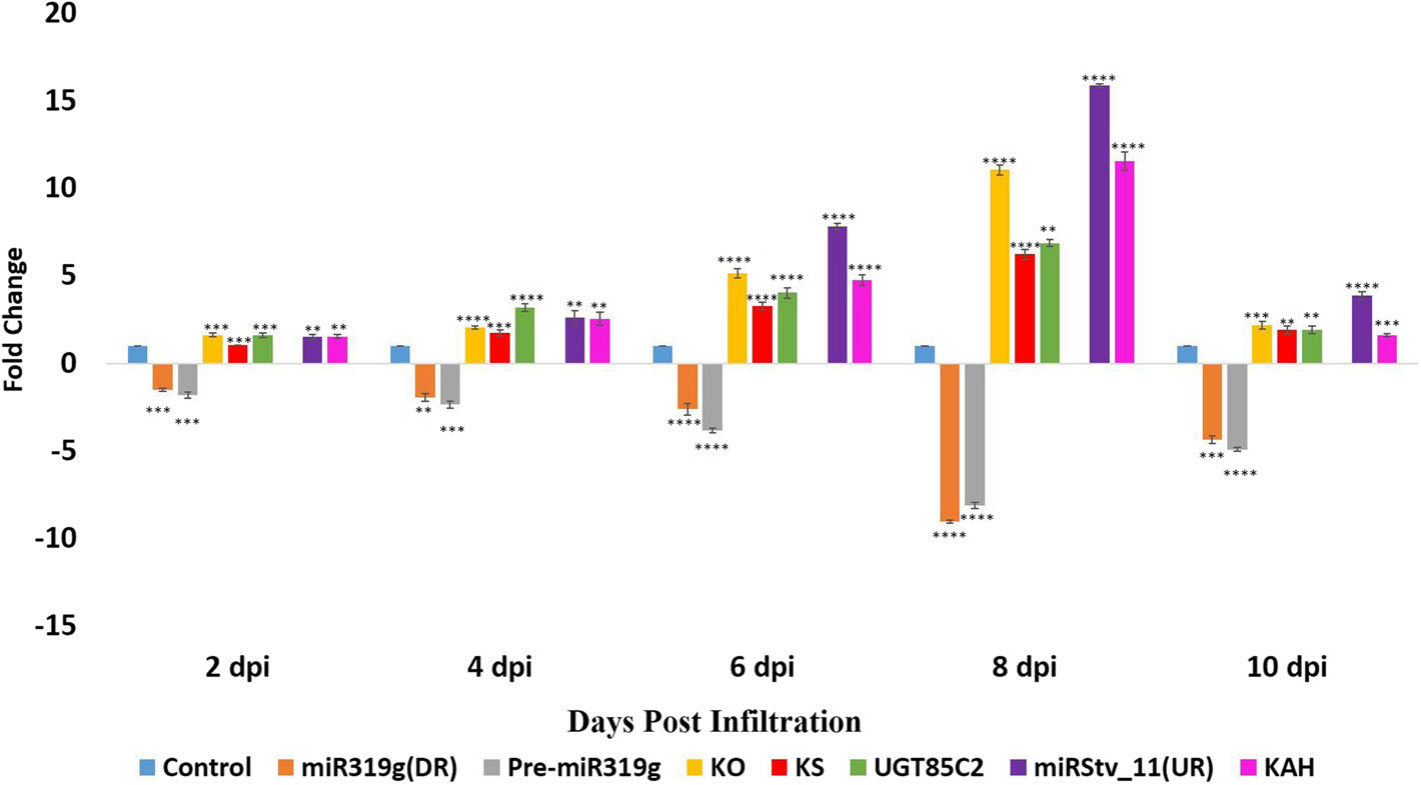
Real-Time PCR analysis of miR319g, miRStv_11 and their target genes when leaves were transformed with miR319g down-regulation and miRStv_11 over-expression constructs. Expression was normalised to that of actin. miRNAs and their target gene levels from the wild type were set as the control. Error bars indicate ±SE (*n* = 3), (*) indicates significance of variance (1*-0.05; 2*-0.01; 3*-0.001; 4*-0.0001). *UR: Up-regulation. *DR: Down-regulation

In order to achieve the over-expression of *KO* (*Kaurene oxidase), KS (Kaurene synthase)* and *UGT85C2 (UDP-glycosyltransferase 85C2)* and *KAH (Kaurenoic acid-13-hydroxylase)* genes together, anti-miR319g and miRStv_11 were co-expressed by mixing the cultures of *Agrobacterium* harbouring anti-miR319g and miRStv_11 in the ratio of 1:1, respectively. This culture was then used to transform leaves of *S. rebaudiana*by agroinfiltration and leaf samples were collected at different days post infiltration as described above. Here, mature and precursor miR319g were found to have up to 9 and 8 fold down-regulation, respectively, while miRStv_11 was found to have up to 15 fold up-regulation in due time of agroinfiltration. This resulted in up to 11, 6.2, 6.8 and 11.5 fold increase in the expression of *KO* (*Kaurene oxidase), KS (Kaurene synthase), UGT85C2 (UDP-glycosyltransferase 85C2)* and *KAH (Kaurenoic acid-13-hydroxylase)* genes, respectively (Fig. 4).

### Profiling of steviol glycosides contents in the leaves of *Stevia rebaudiana* transformed with different constructs

**Fig. 5:**
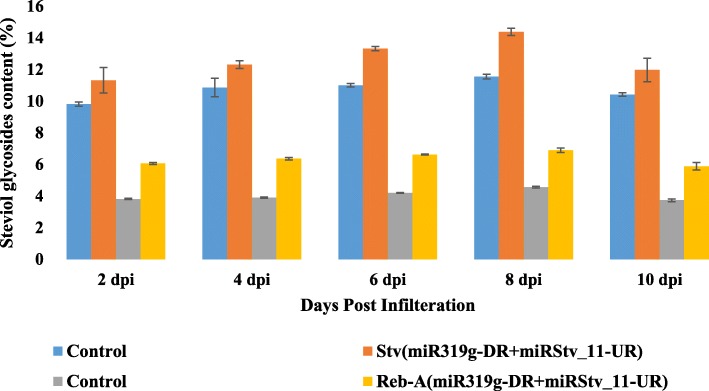
Analysis of steviol glycosides content when leaves were transformed with miR319g down-regulation and miRStv_11 over-expression constructs. Steviol glycosides from the wild type were taken as control. Error bars indicate ±SE (*n* = 3), (*) indicates significance of variance (1*-0.05; 2*-0.01; 3*-0.001; 4*-0.0001). *UR: Up-regulation. *DR: Down-regulation

**Fig. 6:**
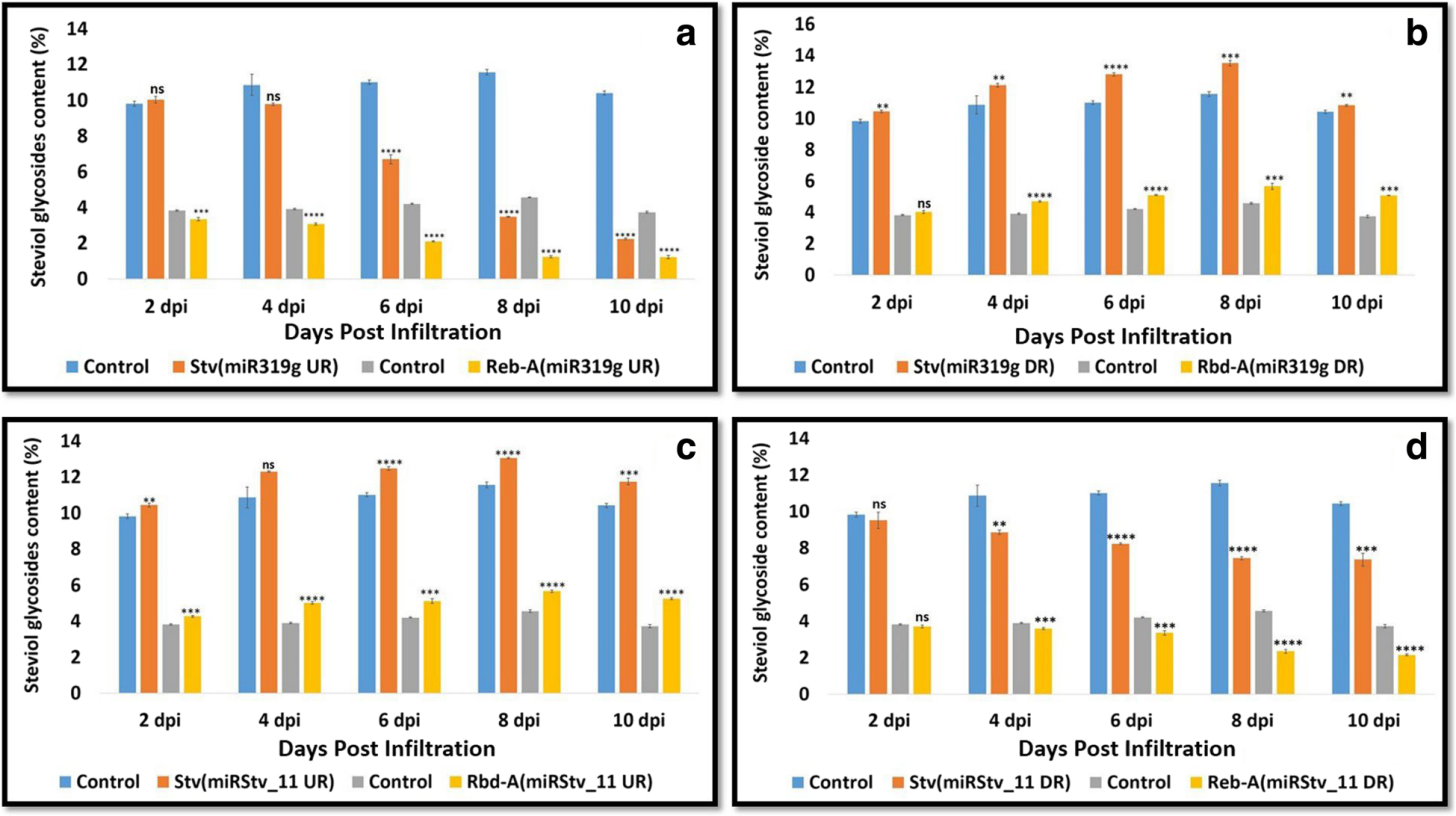
Analysis of steviol glycosides content when leaves were transformed with **(a)** miR319g over-expression construct; **b** miR319g down-regulation construct; **c** miRStv_11 over-expressionconstruct; **d** miRStv_11 down-regulation construct. Steviol glycosides from the wild type were taken as control. Error bars indicate ±SE (*n* = 3), (*) indicates significance of variance. *UR: Up-regulation. *DR: Down-regulation

Leaves of *S. rebaudiana* transformed with different miRNA constructs (miR319g, miRStv_11, anti-miR319g, anti-miRStv_11 and anti-miR319g-miRStv_11) showed a significant (*p ≤ 0.05*) increase in stevioside and rebaudioside-A contents relative to that of the control. The maximum stevioside content, 143.88 mg/gm leaf dry weight was obtained, when leaves were transformed with both anti-miR319g and miRStv_11 together. The increase was about 24.5% as compared to the control. Enhancement was also observed in the rebaudioside-A content, which increased to 68.99 mg/gm leaf dry weight, i.e. 51% increase as compared to the control (Fig. 5). Leaves, when transformed with miR319g and miRStv_11 individually resulted in 69.9% decrease, while 12.9% increase in stevioside; and 72.5% decrease, while 22.8% increase in rebaudioside-A content, respectively (Fig. 6). In another experiment, over expression of anti-miR319g and anti-miRStv_11 resulted in 16.96% increase, while 35.67% decrease in stevioside; and 19.43% increase, while 19.22% decrease in rebaudioside-A content, respectively (Fig. 6).

The sweetening property of *S. rebaudiana* was also examined by calculating the ratio of rebaudioside-A to stevioside. In the present study, a significant difference (*p ≤ 0.05*) was observed in the ratio of rebaudioside-A to stevioside, when the leaves of *S. rebaudiana* were transformed with different miRNA constructs. The ratio of rebaudioside-A to stevioside increased from 0.39 (control plant) to 0.47, 0.42 and 0.41 for leaves transformed with anti-miR319 and miRStv_11 together, and individually with miRStv_11 and miR319g, respectively. On the other hand, the rebaudioside-A to stevioside ratio was found to decrease from 0.39 (control plant) to 0.36 and 0.31, when the leaves were transformed with miR319g and anti-miRStv_11, respectively.

## Discussion

In light of the fact that despite the ever increasing prevalence of diabetes and obesity, we are still in search of a definitive cure of the either condition. Consequently, diabetic and obesity diagnosed individuals must resort to lifestyle and diet changes in order to effectively manage and curtail their condition. This has transitioned from being mere advice to a need of the hour.

*Stevia rebaudiana* is a plant that presents enormous scope to the management of diabetes and obesity having already widespread use in a variety of global products, the most common being a sugar substitute, especially due to the fact that artificial sweeteners are wrapped in controversy over harmful side effects. The biggest highlight of stevia as a sugar substitute is its zero-calorie content and negligible side effects.

In order to facilitate more widespread usage of stevia in commercial products, there is a need to make it palatable. Metabolic engineering may further pave a way to improve the yield of steviol gycosides and therefore, the quality of the natural sweetener by increasing the contents of these metabolites in this plant. One of the ways to tinker with biosynthetic pathway is through modulating miRNA levels. In our previous study, we for the first time identified miRNAs associated with genes of steviol gycosides biosynthetic pathway in *S. rebaudiana.* Among all the miRNAs analysed for their expression levels, we found the two (miR319g and miRStv_11) which showed the expression patterns similar to that of their target mRNAs. On the other hand, the other miRNAs were found to have expression pattern opposite to their target mRNAs [28]. Thus, it became quite interesting to understand how these two miRNAs (miR319g and miRStv_11) are involved in regulation of steviol glycosides biosynthetic pathway genes. In the present study, we characterize the function of these miRNAs. For this, we over-expressed miR319g and miRStv_11 in leaves of *S. rebaudiana* plant by syringe agroinfiltration method [5, 31] and the samples were collected after 2, 4, 6, 8 and 10 days post infiltration. Infiltration of over-expression construct corresponding to miRStv_11 was found to significantly up-regulate the expression of its target gene, *KAH* up to 8.16 fold than the control. Similar results, where miRNAs were found to induce gene expression, were also reported in earlier studies [11, 23]. Place et al. in 2007 suggested that miRNA induced gene expression involves miRNA directly binding to complementary DNA within gene promoters to trigger gene expression. In this regard, miRNA functions like a transcription factor targeting complementary motifs in gene promoters. In our study too, we found the target sites of miRStv_11 on the promoter region of *KAH* gene. To further check the binding of miRStv_11 with the promoter region of *KAH* gene, docking of the two was done by three dimentional structure modelling. High negative value (− 937.59) of desolvation energy indicates high feasibility of the reaction and thus strong binding affinify of miRStv_11 with the *KAH* gene promoter region. This prompted us to speculate that miRStv_11 complementary to sequence in *KAH* promoter may induce gene expression as earlier reported by Place et al. in 2007. When the effect of enhancement of *KAH* expression under the influence of over-expression of miRStv_11 was checked on steviol gycosides content, it was found to be 130.49 mg/g leaf dry weight for stevioside, which was 12.9% higher than that of the control, and 57 mg/g leaf dry weight for rebaudioside-A, which was 22.8% higher than the control. To check the sweetness indices of the plant, the ratio of rebaudioside-A to stevioside was also calculated and it was found to increase from 0.39 (control) to 0.42. Thus, the data suggest that *KAH* is the rate limitinggene involved in steviol gycosides biosynthesis and greatly influence steviol gycosides contents. Similar results, where *KAH* gene has been documented to successfully synthesize steviol in prokaryotic expression systems have been documented in earlier reports [7, 32]. Further, another independent study has documented the role of this gene in regulation of steviol gycosides content in *S. rebaudiana* [31].

**Fig. 7:**
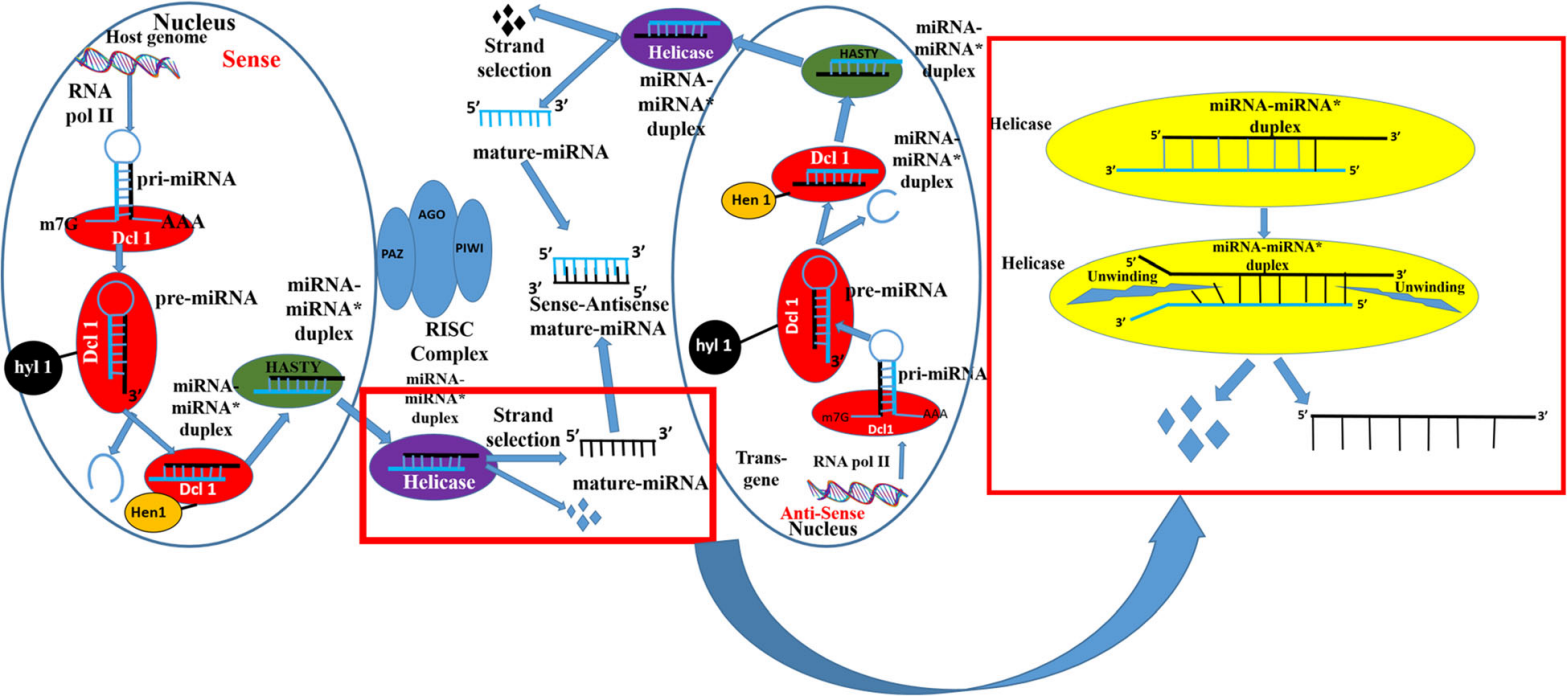
Pictorial depiction of miRNA interference approach involves processing of precursor anti-miRNA within the host genome in a similar manner as of the native miRNA. In this regard, the passenger strand of miRNA which now, is the guide strand of anti-miRNA remain functional thus, binding to the guide strand of miRNA thus making it unavailable to the RISC complex and hence resulting in knock-down of target miRNA

**Fig. 8:**
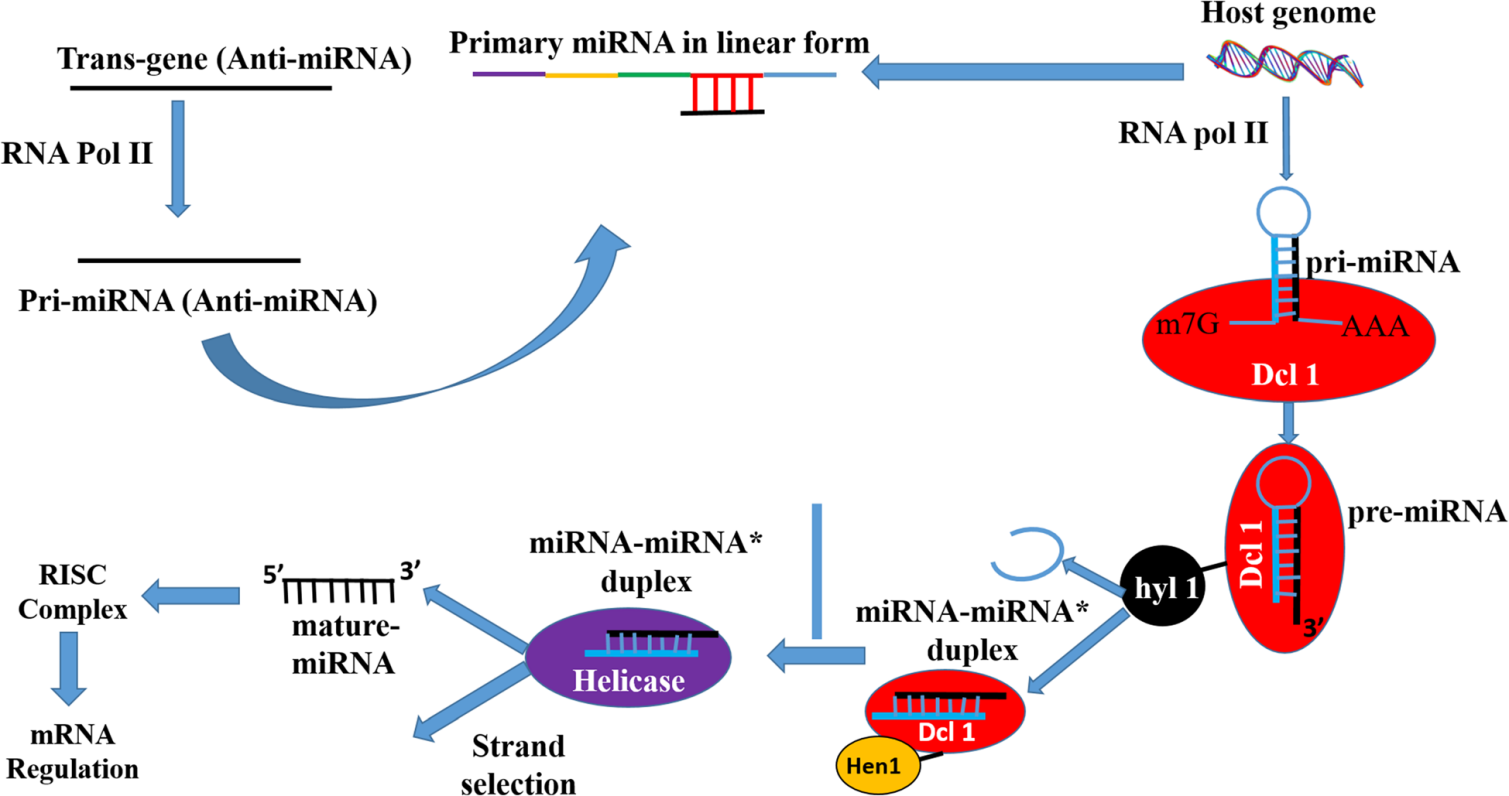
Pictorial depiction of possible mechanism for down-regulation of precursor miRNAcould be that the antisense precursor formed after processing of anti-miRNA must be binding with the complimentary regions to the primary transcript of the miRNA gene, thus inhibiting its further processing and hence the level of precursor miRNA

When the over-expression construct corresponding to miR319g was infiltered in leaves of *S. rebaudiana*, it was found to significantly down-regulate the expression of its target genes, i.e. *KO, KS* and *UGT85C2.* The maximum down-regulation of up to 11.17 fold was found in *KO* transcript suggesting it as a potential target of miR319g. The effect of down-regulation of these genes on steviol gycosides content was also observed. Similar kinds of regulatory roles of miRNAs have also been reported by other investigators, where miRNAs down-regulate the expression of their target genes as a result influencing the synthesis of metabolites [21, 26]. Since miR319g showed negative effect on *KO, KS* and *UGT85C2* genes which are directly involved in steviol gycosides biosynthetic pathway. Hence, down-regulation of their expression by miR319g reduces steviol gycosides contents and rebaudioside-A to stevioside ratio. It was therefore, important to antagonise the repressive action of miR319g in order to achieve high steviol gycosides contents and rebaudioside-A to stevioside ratio. One of the indispensable approaches to antagonize miRNA function could be inhibition or loss of the function of the target miRNA. There are various reports, which documents the inhibition of target miRNAs by modified antisense oligonucleotides in animal and human cell lines [13, 15, 18]. Further, He et al. in 2016, reported miRNA knockdown by sucrose mediated delivery of chemically modified antisense oligonucleotides to rice protoplast and intact leaves resulted in efficient inhibition of miRNAs with concurrent de-repression of their target genes [8]. It has been demonstrated that AMOs need to be chemically modified to improve their functional potency and to provide protection against nuclease degradation [16]. Modifications that increase binding affinity also reduce binding specificity, especially when oligonucleotide concentrations are high [16]. However, in plant miRNAs specificity plays an important role as they recognize target mRNAs mainly through perfect or nearly perfect base pairing [2]. Therefore, a logical approach of silencing plant miRNAs is to use antisense precursor of target miRNA. Thus, antisense precursors of both miR319g and miRStv_11 were used to silence their respective target miRNAs and their impact on their genes as well as on steviol glycosides contents were evaluated. When anti-miR319g and anti-miRStv_11 were over-expressed and their effects were analysed on the expression of the target genes of these miRNAs, 7.24, 5.29, 6.22 fold de-repression of *KO, KS, UGT85C2* genes and 8.15 fold repression in *KAH* gene, respectively were observed. He et al. in 2016 has documented similar results, where inhibition of miRNA resulted in de-repression of its target gene [8], while Place et al., in 2008 has reported a case, where knock-down of miR373 resulted in repression of its target gene [23]. Although, the exact mechanism of miRNA interference remain unclear, but it is known that anti-miRNA binds complimentary miRNA and selectively block its action on the target gene [13, 15, 18]. Thus, a simple model for the mechanism of anti-miRNA induced knock-down of its target miRNA involves processing of precursor anti-miRNA within the host genome in a similar manner as of the native miRNA. In this regard, the passenger strand of miRNA, which now is the guide strand of anti-miRNA remain functional thus, binding to the guide strand of miRNA making it unavailable to the RISC complex and hence, resulting in knock-down of miRNA (Fig. 7). To confirm that it is the sense strand of anti-miRNA, which remains functional, we calculated the thermodynamic stability of both the strands of anti-miRNAs as well as miRNAs. It has been reported that the strand which remains functional among the mature miRNA-miRNA* duplex will always be the one whose 5′ end is less tightly paired to its complement [30]. The internal stability of both sense and antisense strands of anti-miRNAs as well as miRNAs were calculated according to the nearest neighbour method as described in previous reports [4, 12]. To calculate the internal stability i.e. 5′ instability, the hexameric sub sequences were taken, because the sitting site of helicases is six base pair [22, 25]. The sense strand of miR319g and miRStv_11 as well as anti-miR319g and anti-miRStv_11 were found to have higher free energy (− 2.3, − 2.8 and − 1.3, − 1.9 Kcal/mol) than that of the anti-sense strand (− 3.7, − 4.8 and − 3.6, − 3.2 Kcal/mol), thus making it more unstable and hence, functional or guide strand. The presence of antisense strands in leaves transformed with anti-miRNAs were also confirmed by stem-loop RT-PCR and the bands corresponding to 50 bp ladder obtained thus, supports our data for strand selection as well as for miRNA interference. To check, if the miRNA interference occurs only at mature miRNA level or it has effect on precursor level too, we analysed the expression of precursors of miR319g and miRStv_11 in leaves transformed with anti-miR319g and anti-miRStv_11, respectively. And as expected, the levels of precursors of miR319g and miRStv_11 were found to decrease up to 8.28 and 8.37 fold, respectively in due course of agroinfiltration. The possible mechanism for this down-regulation could be that the antisense precursor formed after processing of anti-miRNA must be binding with the complimentary regions to the primary transcript of the miRNA gene, thus inhibiting its further processing and hence, the level of precursor miRNA (Fig. 8). Further, since the target genes of both miR319g and miRStv_11 were directly involved in steviol gycosides biosynthetic pathway and their over-expression/down-regulation was having significant effect on steviol glycosides content therefore, in order to enhance all the four target genes, viz. *KO, KS, UGT85C2* and *KAH*, we co-expressed anti-miR319g and miRStv_11. This resulted in enhancement of all the four genes up to 11.05 (*KO*), 6.21 (*KS*), 6.85 (*UGT85C2*) and 11.54 (*KAH*) fold. When the effect of up-regulation of these genes on steviol glycosides content was analysed, it was found to be 143.88 and 68.99 mg/gm leaf dry weight for stevioside and rebaudioside-A, respectively which was 24.5 and 51% higher than the control. Rebaudioside-A to stevioside ratio was also found to increase from 0.39 (control) to 0.47. Similar kind of reports, where co-expression of miRNAs or genes influenced the metabolite contents, have been documented previously [24, 25].

## Methods

### Plant material

Potted plants of *S. rebaudiana*bertoni, cv. CIM-Mithi, procured from the Central Institute of Medicinal and Aromatic Plants (CIMAP), Pantnagar, India and maintained under greenhouse conditions at Jamia Hamdard (Hamdard University, New Delhi, India) at a temperature of 25 ± 2 °C and relative humidity of 60–70%, were used for the study. *S. rebaudiana*cv. CIM-Mithi is a registered variety of Central Institute of Medicinal and Aromatic Plants (CIMAP), a national laboratory of Council of Scientific and Industrial Research (CSIR), Government of India. The variety is maintained in the Field Gene bank of CSIR-CIMAP for multiplication and cultivation purpose [14]. Leaves were collected at the pre-flowering stage from *S. rebaudiana* plants maintained under greenhouse conditions for RNA isolation and conducting over-expression studies.

### Synthesis of miRNA over-expression constructs

The miRNA319g and miRStv_11 were isolated from leaves of *Stevia rebaudiana* through Polymerase Chain Reaction (PCR) amplification using genomic DNA as a template. Primers for miR319g and miRstv_11 were designed based on the published sequence of miRNA319g from *Populus trichocarpa* (MIPF000001) in the existing miRbase (database of micro-RNA sequences) and stevia EST sequences (BG524443.1) as submitted in NCBI, respectively. The forward and reverse primers were supplemented at 5′ end with the restriction site *Bgl*II and *BstE*II for directional cloning, respectively. Sequences of the miRNA primers are summarized in Additional file 3. The amplified miRNA precursors of miR319g and miRStv_11 were individually cloned into pGEMT easy vector. Cloned sequences were sent for sequencing and then sequences were analysed by ClustalW and NCBI BLAST. The resulting pGEMT easy vectors were restriction digested to fish out miRNA precursor fragments. The fragments of miR319g and miRStv_11 were then sub cloned in sense orientation in pCAMBIA 1301 in place of GUS intron. The over-expression constructs were under the control of constitutively expressing 35S cauliflower mosaic virus promoter. The prepared constructs were confirmed by double digestion with restriction enzymes *Bgl*II and *BstE*II. Fishing out of fragments of size 196 and 320 bp on double digestion validated the preparation of over-expression constructs corresponding to miR319g and miRStv_11, respectively. The prepared over-expression constructs were finally transformed into *Agrobacterium* strain EHA105. Syringe mediated agroinfiltration method was adopted to characterize the function of these miRNAs. The culture was gently injected into the leaf to prevent leaf damage. The movement of culture was visualized with naked eye. The success of infiltration was estimated by the visible zone of infiltration present on the leaf surface.

### Synthesis of miRNA knock-down constructs

For inhibition of miR319g and miRStv_11, anti-miR319g and anti-miRStv_11 knock-down constructs were synthesised, respectively by cloning precursor antisense of target miRNAs in expression vector. To generate miRNA precursors of miR319g and miRStv_11 in antisense orientation, the restriction sites of *BstE*II and *Bgl*II were added to the forward and reverse primers at 5′ ends, respectively. Primer designing and PCR amplification of anti-miRNAs was similar as described above for miR319g and miRStv_11. Sequences of the anti-miRNA primers are summarized in Additional file 3. The amplified antisense miRNA precursors of miR319g and miRStv_11 were individually cloned into pGEMT easy vector. The resulting pGEMT easy vectors were restriction digested to fish out antisense miRNA precursor fragments. The fragments of 196 and 320 bp precursors of miR319g and miRStv_11, subcloned in antisense orientation in pCAMBIA 1301 in place of GUS intron. The knock-down constructs were under the control of constitutively expressing 35S cauliflower mosaic virus promoter. The validation of prepared constructs was done by double digesting them with restriction enzymes *Bgl*II and *BstE*II. The fragments of 196 and 320 bp thus confirmed the preparation of knock-down constructs corresponding to anti-miR319g and miRStv_11, respectively. The prepared knock-down constructs were finally cloned into *Agrobacterium* strain EHA105. Syringe mediated agroinfiltration method was adapted to transform *S. rebaudiana* for characterization of the function of anti-miR319g and anti-miRStv_11.

### Thermodynamic profiles of guide and passenger strands of miRNAs and anti-miRNAs

To determine strand functionality of the mature miRNA-miRNA* duplex, 5′ instabilities of both the strands were calculated for miRNAs as well as anti-miRNAs, using nearest neighbour method [4]. The internal stability values reflects the stability of hexamer sub sequences within the sequence under investigation. No initiation ∆G values were included in calculations. To simplify calculations, the internal stability values were first calculated for the perfect duplex and then the mismatch penalty and any penalty for bulges/loops were included, wherever necessary. Mismatch penalties varied on the nature of the eliminated base pair and were calculated according to the nearest neighbour rules [12].

To support this mathematical data, stem-loop RT-PCR was done to validate the selection of anti-miR319g and anti-miRStv_11 strands in leaves of *S. rebaudiana,* transformed with knockdown constructs. For this, stem loop RT primers specific to antisense strands were designed and allowed to bind to the antisense miRNA and reverse transcribed in a pulsed RT reaction using RevertAid™ H Minus cDNA synthesis kit (Fermentas, USA). Cycling conditions for cDNA synthesis were kept as described by Gasic et al. [34]. The RT product (cDNA) synthesized was then used as a template to amplify the anti-miR319g and anti-miRStv_11 sequences through semi-quantitative RT-PCR by using anti-miR319g and anti-miRStv_11 specific forward primer and universal reverse primer, respectively. Sequences of the anti-miRNA primers are summarized in Additional file 3.

### *Agrobacterium* mediated transient transformation

The prepared miR319g and miRStv_11 over-expression as well as knock-down constructs were used to transform *Agrobacterium tumefaciens* strain EHA105. The transformed *Agrobacteria* were initially grown in 5 ml of liquid YEM supplemented with rifampicin (10 mg/ml) and kanamycin (50 mg/ml) overnight at 28 °C and 200 rpm. Two ml of cultured cells were pelleted down at 1000 g for 10 min at room temperature and re-suspended in 1 ml of infiltration medium [250 mg D-glucose, 20 mM MES (2-(N-morpholino) ethane sulfonic acid), 2 mM Na_3_PO_4_.12H_2_O (trisodium orthophosphate; BDH), 1 mM acetosyringone]. This step was repeated twice. Absorption was taken at 600 nm (0.1 OD). Syringe agroinfiltration method was used for inoculation of *Agrobacterium*, where the processed culture was taken in 1 ml syringe and tip of the syringe was placed against the underside of the leaf over the needle mark and pressed down gently on the plunger, while directly supporting the upper side of the leaf with the finger. At optimal OD_600,_ the over-expression and knockdown constructs were first separately inoculated to check their individual effect on miRNAs and mRNAs level. Further, on the basis of results obtained and in order to achieve the over-expression of all the four genes (*KO, KS, UGT85C2* and *KAH)* of the steviol glycosides biosynthetic pathway, anti-miR319g and miRStv_11 were co-transformed in the leaves of *S. rebaudiana*. For this, the cultures of *Agrobacterium* harbouring anti-miR319g and miRStv_11 were mixed in the ratio of 1:1. The infiltrated leaf samples were collected on 2, 4, 6, 8 and 10 days post infiltration (dpi). The collected leaf samples were employed for further molecular and chromatographic analysis.

### Total RNA extraction and expression profiling of miRNA and mRNA levels

The total RNA samples were isolated from leaves of *S. rebaudiana*at pre-flowering stage transformed with different over-expression constructs, using RNeasy plant mini kit (Qiagen) according to the manufacturer’s instructions. For quantification of mature miRNAs*viz.* miR319g and miRStv_11, specific cDNAs were synthesized using RevertAid™ H Minus cDNA synthesis kit (Fermentas, USA). Cycling conditions for cDNA synthesis were kept as described by Gasic et al. [34]. Following reverse transcription, quantitative real time PCR (qPCR) was performed in triplicate, using SYBR green chemistry ([3] on a real-time thermal cycler (Light Cycler 480, Roche, USA). β-actin was used as an internal control. Reaction mixture and cycling conditions were same as described in our previous report [28]. For amplification of precursor miRNAs and target mRNAs, RT-PCR was carried out using two step RT-PCR kit (Qiagen) with precursor miRNA and mRNA specific forward and reverse primers, respectively.

Fold variations in expression of miRNAs and mRNAs between RNA samples were calculated using ∆∆C_T_ method [17]. For a transformed sample, fold change relative to the control sample was calculated as 2^-∆∆CT^, where ∆∆C_T_ = (C_T_ of transformed sample-C_T_ of β-actin)-(C_T_ of control sample-C_T_ of β-actin). The standard deviation and standard error were calculated from three replicates and the level of significance was measured through student’s t-test at the level of *p* ≤ 0.05.

### Extraction and quantification of steviol glycosides content

Leaves of *S. rebaudiana* transformed with different constructs, were collected and dried to a constant weight in dark at 25 °C and ground to a fine powder in a mortar-pestle All the samples were then used for the estimation of stevioside and rebaudioside-A as described in our previous study [27].

### Bio-informatic prediction of binding affinity of miRStv_11 with promoter region of *Kaurenoic acid hydroxylase* gene

#### Three dimensional structure modeling

To model three dimensional structures of the biomolecules, two softwares were used. Make-na server (http://structure.usc.edu/make-na/server.html) was used to model gene B-DNA 3D structure of miRNA interaction site at the gene. It is a web-based utility to easily generate linear DNA and RNA models using Nucleic Acid Builder. It’s a flexible tool, which provides both the B- form as well as A- form of DNA. ModeRNA ((http://genesilico.pl/moderna/) was used to model 3D structure of miRNA (miRStv_11). It is a program for comparative modeling of RNA 3D structures.

#### Interaction of promoter regions and miRNA

To investigate miRNA-DNA interactions, docking study was performed with the help of web-server PatchDock (https://bioinfo3d.cs.tau.ac.il/PatchDock/). The PatchDock first divide surface of the molecules into concave, convex and flat patches, then matches complementary patches to generate candidate transformations. The transformations are evaluated by a scoring function which involves atomic and geometric desolvation energy. The algorithm of the software has three major steps; (i) Molecular Shape Representation: compute the molecular surface of the molecule, (ii) Surface Patch Matching: match the patches detected in the first step by applying geometic hashing and Pose-Clustering, and (iii) Filtering and Scoring: to find best docked candidates, which are ranked according to a geometric shape complementarity score.

## Conclusion

In summary, we successfully validated the role of miR319g and miRStv_11 in regulating the genes of steviol glycosides biosynthetic pathway and their effect on steviol glycosides contents. Our results, revealed the positively correlated miRNA-mRNA interaction network in plants, where miRStv_11 enhanced the expression of *KAH* gene by targeting specific sites in gene promoter region. We also successfully silenced the expression of miRNAs using their antisense precursors. Overall, our study thus reveals the more complex nature and fundamental importance of miRNAs in biosynthetic pathway related gene networks and hence, these miRNAs can be successfully employed to enhance the ratio of rebaudioside-A to stevioside, thus enhancing the sweetening indices of this plant and making it more palatable.

## Additional files

Additional file 1: Structure of miRStv_11 obtained from *S. rebaudiana* using mfold. (PDF 193 kb)

Additional file 2: Structure of miRStv_11 obtained from *S. rebaudiana* using mfold. (PDF 205 kb)

Additional file 3: Primer sequences and their annealing conditions. (PDF 175 kb)

Additional file 4: Atomic level interactions of miRNA and miRNA binding sites. miRStv_11 bases are shown in three letters and KAH gene promoter region bases are depicted in one letter codes. (PDF 175 kb)

Additional file 5: Sequences of miR319g and miRStv_11 obtained from *Stevia rebaudiana*. (PDF 196 kb)

## Data Availability

All data generated or analysed during this study are included in this published article (and its supplementary information files).

## Abbreviations

DR: Down-regulation
*KAH*: *Kaurenoic acid-13-hydroxylase*
*KO*: *Kaurene oxidase*
*KS*: *Kaurene synthase*
*UGT85C2*: *UDP-glycosyltransferase 85C2*
UR: Up-regulation
