# Supplementary material for: Co-expression of anti-miR319g and miRStv_11 lead to enhanced steviol glycosides content in Stevia rebaudiana
Source: BMC Plant Biol. 2019 Jun 24;19:274. doi: 10.1186/s12870-019-1871-2 (PMC6591970; doi:10.1186/s12870-019-1871-2)
Supplement: Supplementary file 3 — Primer sequences and their annealing conditions. (PDF 175 kb) [file 12870_2019_1871_MOESM3_ESM.pdf]

Additional file 3: Primer sequences and their annealing conditions

miRNA	Primer Sequence		Annealing conditions
miR319g (Precursor Sequence)	FP	AGATCTGAGCTTCTTTCAGCCCACTC	54.3°C, 1.30min
	RP	GGTAACCAAGCAGCTCCCTTCAGTCCAA	
miRStv_11 (Precursor Sequence)	FP	AGATCTCAGGTCTTGGCAACTACATCTC	54.3°C, 1.30min
	RP	GGTAACCGTGCACCCTGTTGGTAGTAAG	
anti-miR319g (Precursor Sequence)	FP	GGTACCGAGCTTCTTTCAGCCCACTC	54.3°C, 1.30min
	RP	AGATCTAAGGAGCTCCCTTCAGTCCAA	
anti-miRStv_11 (Precursor Sequence)	FP	GGTACCCAGGTCTTGGCAACTACATCTC	54.3°C, 1.30min
	RP	AGATCTGTGCACCCTGTTGGTAGTAAG	
Anti-miRstv_11 (Mature miRNA Sequence)	RTP	GTCGTATCCAGTGCAGGGTCCGAGGTATTCGCACTGGATACGACGGCTCA	55°C, 30sec
	FP	CGCGAUGACUCGUCAUUU	
Anti-miR319g (Mature miRNA Sequence)	RTP	GTCGTATCCAGTGCAGGGTCCGAGGTATTCGCACTGGATACGACTTGGAC	55°C, 30sec
	FP	GCGCATGAGCTCCCTTCA	
URP		GTGCAGGGTCCGAGGT	
