# Supplementary material for: Co-expression of anti-miR319g and miRStv_11 lead to enhanced steviol glycosides content in Stevia rebaudiana
Source: BMC Plant Biol. 2019 Jun 24;19:274. doi: 10.1186/s12870-019-1871-2 (PMC6591970; doi:10.1186/s12870-019-1871-2)
Supplement: Supplementary file 4 — Atomic level interactions of miRNA and miRNA binding sites. miRStv_11 bases are shown in three letters and KAH gene promoter region bases are depicted in one letter codes. (PDF 175 kb) [file 12870_2019_1871_MOESM4_ESM.pdf]

**Additional file 4: Atomic level interactions of miRNA and miRNA binding sites. miRStv_11 bases are shown in three letters and *KAH* gene promoter region bases are depicted in one letter codes.**

| **Donor** | **Acceptor** | **Donor Acceptor Distance** |
|---|---|---|
| C 3.B N4 | GUA 13.C N3 | 3.3 |
| A 5.B N6 | ADE 14.C OP1 | 2.2 |
| A 6.B N6 | ADE 14.C N7 | 2.9 |
| C 8.B N4 | GUA 3.C O6 | 2.9 |
| C 8.B N4 | URA 16.C O4 | 3.5 |
| C 9.B N4 | GUA 2.C O6 | 2.6 |
| C 10.B N4 | GUA 1.C O6 | 3.4 |
| C 10.B N4 | URA 19.C O4 | 3.6 |
| ADE 14.C N6 | A 6.B N7 | 2.6 |
| ADE 14.C N6 | A 6.B O2P | 3.3 |
| CYT 17.C N4 | G 2.A O6 | 2.9 |
| CYT 17.C N4 | G 3.A O6 | 3.6 |
| ADE 5B.C N6 | A 6.B O5' | 2.5 |
