# Supplementary material for: Co-expression of anti-miR319g and miRStv_11 lead to enhanced steviol glycosides content in Stevia rebaudiana
Source: BMC Plant Biol. 2019 Jun 24;19:274. doi: 10.1186/s12870-019-1871-2 (PMC6591970; doi:10.1186/s12870-019-1871-2)
Supplement: Supplementary file 1 — Structure of miRStv_11 obtained from S. rebaudiana using mfold. (PDF 193 kb) [file 12870_2019_1871_MOESM1_ESM.pdf]

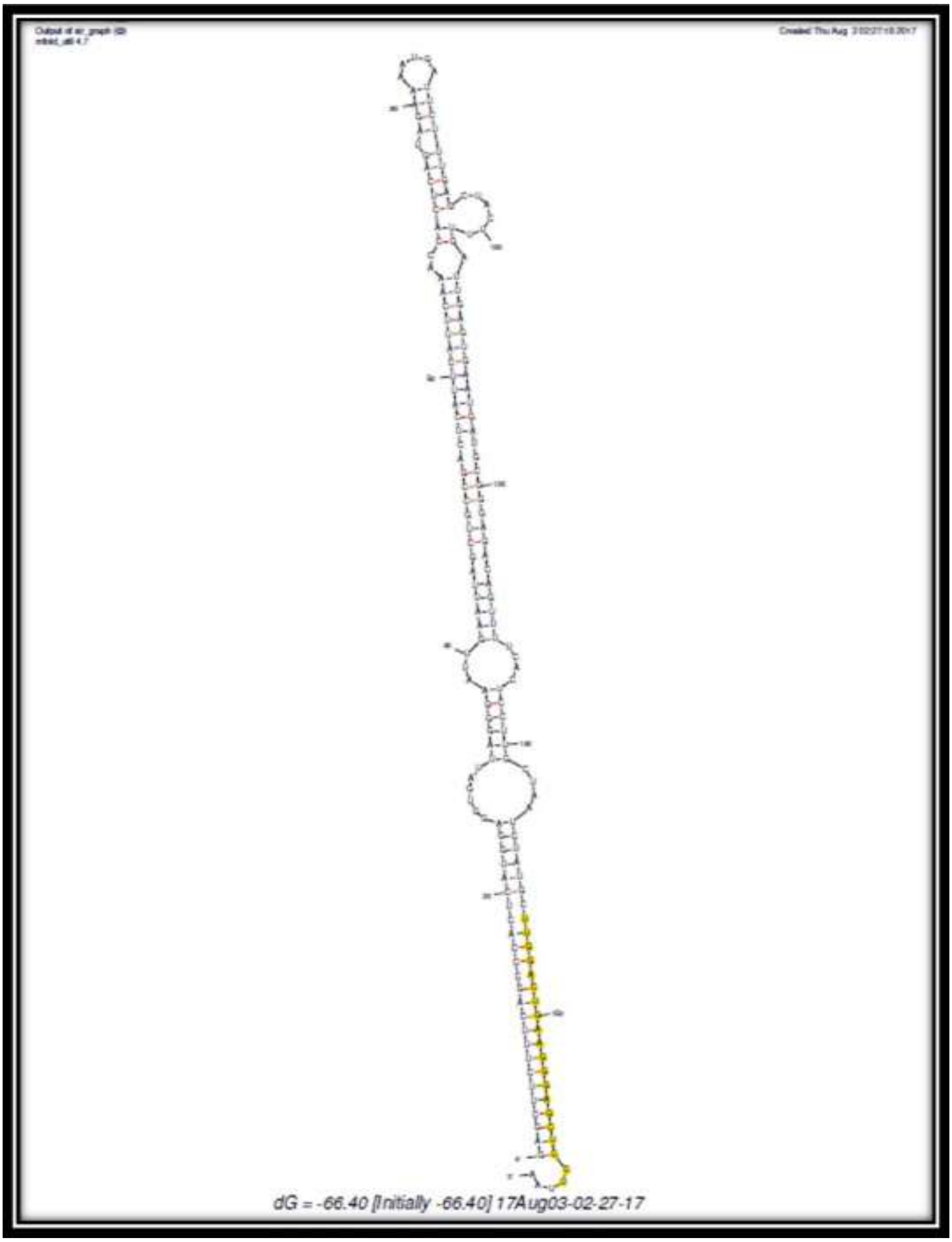

Additional file 1: Structure of miRStv_11 obtained from *S. rebaudiana* using mfold.
