# Supplementary material for: Co-expression of anti-miR319g and miRStv_11 lead to enhanced steviol glycosides content in Stevia rebaudiana
Source: BMC Plant Biol. 2019 Jun 24;19:274. doi: 10.1186/s12870-019-1871-2 (PMC6591970; doi:10.1186/s12870-019-1871-2)
Supplement: Supplementary file 5 — Sequences of miR319g and miRStv_11 obtained from Stevia rebaudiana. (PDF 196 kb) [file 12870_2019_1871_MOESM5_ESM.pdf]

Additional file 5: Sequence of miR319g and miRStv_11 obtained from *Stevia rebaudiana* and their alignment with sequence of miR319g *Populus trichocarpa* and stevia EST, respectively.

miRNA-ID	Sequence
**miR319g**	GAGCTTCTTTCAGCCCACTCATGGAGGTGATTAGGGAATTGAATTAGCTGCCGAC<br>TCATTCATTCAAACCACTCAGTAGAAAATGAGGCGATGTTCTTTGAGCTACTTTGA<br>TTGAGTGAATGATGCGGGAGACAGTTTTCACTCCTTGCTAATCTATGCTTGGACTG<br>AAGGGAGCTCCTTAA
**miRStv_11**	CAGGTCTTGGCAACTACATCTCCGGTGCCATCCTCTTTGAGGAAACTTTGTACCAA<br>TCAACCGTCGAAGGCAAAAAGATCGTAGATATCCTTAATGAACAAGGGATCGTAC<br>CTGGTATCAAGACTGACAAGGTAATATAATGCAATAAAATATAATATAACATTTAT<br>GCATTGAAATTCACAAAATGTTGACTTTGATTTGTCGTTCGGTTCCATTGGCGGGC<br>TCAAATGACGAGTCATGGTGCCAAGGTCTTGATGGGCTAGCCTCGCGCTCGGCA<br>GCTTACTACCAACAGGGTGCAC

1. **Alignment of sequence of miR319g obtained from *S. rebaudiana* with sequence of miR319g obtained from *Populus trichocarpa.***

```
miR319g-Stevia      -------------GAGCTTCTTTCAGCCCACTCATGGAGGTGATTAGGGAATTGAATTAG
miR319g-ptc         TAATCGTGGGAGAGAGCTTCTTTCAGCCCACTCGTGGATAGGACAAAGGGGTTGAACTAG
                                 ********************.**** . ** :*.**..***** ***

miR319g-Stevia      CTGCCGACTCATTCATTCAAACCACTCAGTAGAAAATG---------ATTCTTTGAGCTA
miR319g-ptc         CTGCCGACTCATTCATTCAAGCACTAGTAGAAAAAAAGGCGAACGGTGTTTCTTTTGCTA
                    ********************.*.. : :. *.****:*         .**  ** :****

miR319g-Stevia      CTTTGATTGAGTGAATGATGCGGGAGACAGTTTTCACTCCTTG-CTAATCTATGCTTGGA
miR319g-ptc         CTGTGATTGTGTGAATGATGCGGGAGATAATTTTCCATCCTCTCCTTTTCTGTGCTTGGA
                    ** ******:***************** *.*****..****   **::***.********

miR319g-Stevia      CTGAAGGGAGCTCCTTAA------
miR319g-ptc         CTGAAGGGAGCTCCTTCCTTCTCT
                    ****************..
```

2. **Alignment of sequence of miRStv_11 obtained from *S. rebaudiana* with sequence of stevia EST.**

```
miRStv_11       CAGGTCTTGGCAACTACATCTCCGGTGCCATCCTCTTTGAGGAAACTTTGTACCAATCAA
Stevia-EST      CAGGTCTTGGCAACTACATCTCCGGTGCCATCCTCTTTGAGGAAACTTTGTACCAATCAA
                ************************************************************

miRStv_11       CCGTCGAAGGCAAAAAGATCGTAGATATCCTTAATGAACAAGGGATCGTACCTGGTATCA
Stevia-EST      CTGTCGAAGGCAAAAAGATCGTAGATATCCTTAATGAACAAGGGATCGTACCTGGTATC
                * **********************************************************

miRStv_11       AGACTGACAAGGTAATATAATGCAATAAAATATAATATAACATTTATGCATTGAAATTCA
Stevia-EST      AGACTGACAAGGGTT---------------------------------------------
                ************

miRStv_11       CAAAATGTTGACTTTGATTTGTCGTTCGGTTCCATTGGCGGGCTCAAATGACGAGTCATG
Stevia-EST      --------------------------TGGTTCCATTGGCGGGCTCAAATGACGAGTCATG
                                           *********************************

miRStv_11       GTGCCAAGGTCTTGATGGGCTAGCCTCGCGCTCGGCAGCTTACTACCAACAGGGTGCAC
Stevia-EST      GTGCCAAGGTCTTGATGGGCTAGCCTCGCGCTCGGCAGCTTACTACCAACAGGGTGCAC
                ***********************************************************
```
